# Impact of early relapse within 24 months after first-line systemic therapy (POD24) on outcomes in patients with marginal zone lymphoma: A US multisite study

**DOI:** 10.1186/s13045-023-01448-y

**Published:** 2023-05-08

**Authors:** Narendranath Epperla, Rina Li Welkie, Pallawi Torka, Geoffrey Shouse, Reem Karmali, Lauren Shea, Andrea Anampa-Guzmán, Timothy S. Oh, Heather Reaves, Montreh Tavakkoli, Kathryn Lindsey, Irl Brian Greenwell, Emily Hansinger, Colin Thomas, Sayan Mullick Chowdhury, Kaitlin Annunzio, Beth Christian, Stefan K. Barta, Praveen Ramakrishnan Geethakumari, Nancy L. Bartlett, Alex F. Herrera, Natalie S. Grover, Adam J. Olszewski

**Affiliations:** 1 Division of Hematology, Department of Medicine, Ohio State University Comprehensive Cancer Center, Columbus, OH 43210 USA; 2 Roswell Park Cancer Institute, Buffalo, NY USA; 3 City of Hope, Duarte, CA USA; 4 Northwestern University, Chicago, IL USA; 5 Siteman Cancer Center, Washington University School of Medicine, St. Louis, MO USA; 6 Harold C. Simmons Comprehensive Cancer Center, UT Southwestern Medical Center, Dallas, TX USA; 7 University of Pennsylvania, Philadelphia, PA USA; 8 Hollings Cancer Center, Medical University of South Carolina, Charleston, SC USA; 9 Thomas Jefferson University, Philadelphia, PA USA; 10 Lineberger Comprehensive Cancer Center, University of North Carolina, Chapel Hill, NC USA; 11 Brown University, Providence, RI USA

**Keywords:** POD24, Non-POD24, Marginal zone lymphoma, Overall survival

## Abstract

Progression of disease within 24 months (POD24) from diagnosis in marginal zone lymphoma (MZL) was shown to portend poor outcomes in prior studies. However, many patients with MZL do not require immediate therapy, and the time from diagnosis-to-treatment interval can be highly variable with no universal criteria to initiate systemic therapy. Hence, we sought to evaluate the prognostic relevance of early relapse or progression within 24 months from systemic therapy initiation in a large US cohort. The primary objective was to evaluate the overall survival (OS) in the two groups. The secondary objective included the evaluation of factors predictive of POD24 and the assessment of cumulative incidence of histologic transformation (HT) in POD24 versus non-POD24 groups. The study included 524 patients with 143 (27%) in POD24 and 381 (73%) in non-POD24 groups. Patients with POD24 had inferior OS compared to those without POD24, regardless of the type of systemic therapy received (rituximab monotherapy or immunochemotherapy) at diagnosis. After adjusting for factors associated with inferior OS in the univariate Cox model, POD24 remained associated with significantly inferior OS (HR = 2.50, 95% CI = 1.53–4.09, p = 0.0003) in multivariable analysis. The presence of monoclonal protein at diagnosis and those who received first-line rituximab monotherapy had higher odds of POD24 on logistic regression analysis. Patients with POD24 had a significantly higher risk for HT compared to those without POD24. POD24 in MZL might be associated with adverse biology and could be used as an additional information point in clinical trials and investigated as a marker for worse prognosis.

**Supplementary Information:**

The online version contains supplementary material available at 10.1186/s13045-023-01448-y.

## To the editor

Marginal zone lymphomas (MZL) are a group of indolent B-cell non-Hodgkin lymphomas (NHL) that are classified into three specific subtypes: extranodal MZL of mucosa-associated lymphoid tissue (MALT lymphoma; EMZL), splenic MZL (SMZL), and nodal MZL (NMZL) [1]. For patients requiring treatment, options include single-agent rituximab (R) or R-chemotherapy (immunochemotherapy) with the latter showing a higher rate of complete responses (CR) [2–6]. Progression of disease within 24 months of diagnosis following first-line treatment with rituximab plus cyclophosphamide, doxorubicin, vincristine, and prednisone (R-CHOP) has been shown to portend poor outcomes in FL [7]. Similarly, early relapse after diagnosis in MZL was associated with poor outcomes [8, 9]. However, these studies included only patients requiring immediate therapy for MZL and defined early relapse as lymphoma progression within 24 months (POD24) from diagnosis rather than from the initiation of systemic therapy. In contrast, many patients with MZL do not require immediate therapy [8], and the diagnosis to treatment interval (DTI) may be highly variable with no universal criteria to initiate systemic therapy. Therefore, evaluation of the clinical significance of POD24 in MZL should account for the common scenario of prolonged (DTI). We sought to evaluate the prognostic relevance of early relapse or progression within 24 months from systemic therapy initiation in a large US cohort, without limitation related to preceding DTI.

This multicenter retrospective cohort study included adult patients (18 years or older) with MZL who received first-line treatment on or after 2010 at 11 US medical centers. To be eligible for the analysis, patients must have received systemic therapy in the first-line setting. Patients who never received systemic therapy or those who received only antibiotics, radiation therapy, or surgery were excluded. The study population was divided into two groups designated as POD24 and non-POD24. POD24 was defined as relapse or progression of MZL within 24 months of initiation of systemic therapy. The primary objective of the study was to evaluate the overall survival (OS) in the two groups. OS was defined as the time from the start of first-line systemic therapy until death or last follow‐up. The secondary objective included the evaluation of factors predictive of POD24 and the assessment of the cumulative incidence of histologic transformation in the POD24 versus non-POD24 groups. See the “Additional file 1” for study variables and statistical analysis.

The study included 524 patients. Among these, 143 (27%) were in the POD24 group and 381 (73%) in the non-POD24 group. Table 1 shows the baseline characteristics of the patient population according to POD24 status. To determine the factors independently associated with POD24 (Additional file 1: Table S1), we performed multivariable logistic regression analysis and found that the presence of monoclonal protein at diagnosis was associated with increased odds of POD24 (OR = 2.87, 95% CI = 1.69–4.85, p < 0.0001), while those who received immunochemotherapy had lower odds of POD24 (compared to R, OR = 0.48, 95% CI = 0.29–0.80, p = 0.005, Additional file 1: Table S1).

**Table 1:** Baseline characteristics

Variable	All patients (n = 524) %	POD24 group (n = 143) %	Non-POD24 group (n = 381) %	p-value
Median age, range (yrs)	63 (18–98)	66 (19–98)	62 (18–93)	**0.003**
Sex, n (%)				0.57
Males	268 (51)	76 (53)	192 (50)	
Females	256 (49)	67 (47)	189 (50)	
BMI ≤ 30 kg/m2, n (%)	340 (69)	94 (72)	246 (68)	0.34
MZL subtype, n (%)				**0.04**
NMZL	124 (24)	45 (32)	79 (21)	
SMZL	135 (26)	33 (23)	102 (27)	
EMZL	265 (50)	65 (45)	200 (52)	
ECOG PS, n (%)				**0.03**
0–1	448 (91)	111 (85)	337 (93)	
≥ 2	46 (9)	19 (15)	27 (7)	
Stage, n (%)				0.21
1–2	140 (27)	31 (22)	109 (29)	
3–4	376 (73)	110 (78)	266 (71)	
B symptoms, n (%)	106 (21)	35 (25)	71 (19)	0.12
LDH > ULN, n (%)	135 (27)	46 (36)	89 (24)	**0.01**
Albumin < ULN, n (%)	80 (16)	29 (22)	51 (14)	**0.03**
B2M > ULN, n (%)	129 (52)	35 (53)	94 (52)	0.88
Monoclonal protein, n (%)	151 (32)	62 (47)	89 (27)	** < 0.0001**
BM involvement, n (%)	243 (55)	69 (57)	174 (55)	0.59
Median WBC, K/uL (range)	6.2 (0.7–131)	5.8 (0.7–54.2)	6.3 (1.6 – 131)	0.21
Median Hgb, g/dL (range)	12.5 (3.7–18.9)	12.3 (5.5–15.6)	12.6 (3.7–18.9)	0.20
First-line treatment, n (%)				**0.02**
Rituximab alone	296 (56)	95 (66)	201 (53)	
R-chemotherapy	200 (38)	42 (29)	158 (41)	
Others	28 (5)	6 (4)	22 (6)	

*BMI* body mass index, *ECOG PS* Eastern Cooperative Oncology Group performance status, *LDH* lactate dehydrogenase, *ULN* upper limit of normal, *B2M* beta 2 microglobulin, *BM* bone marrow

**Fig. 1:**
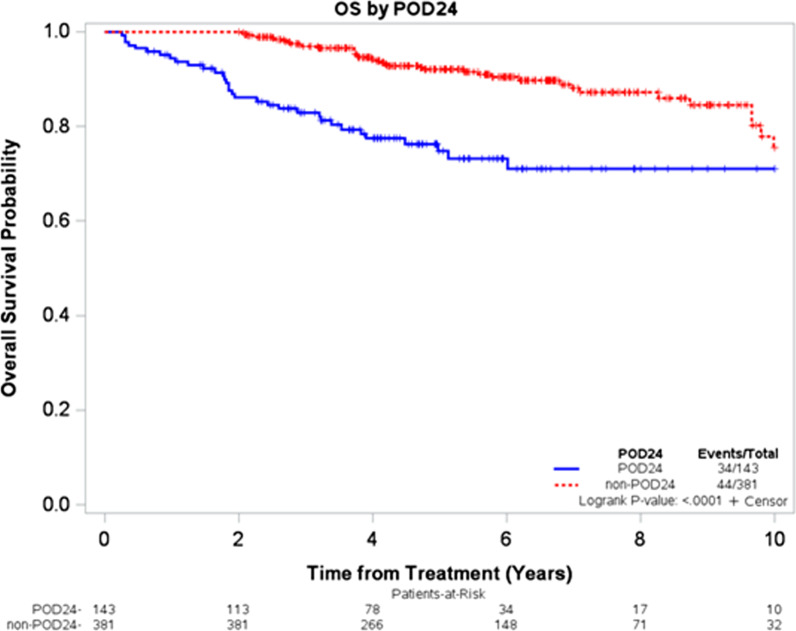
Overall Survival between POD24 and non-POD24 groups

Median follow-up for the entire cohort from the start of first-line therapy was 17.2 years (95% CI, 14.9-NR); 78 patients (15%) died during the follow-up. The median OS was not reached in either group, however, the 3- and 5-year OS estimates were 83% (95% CI, 75–88%) and 75% (95% CI, 66–82%) in the POD24 group compared to 97% (95% CI, 95–98%) and 92% (95% CI, 88–95%) in the non-POD24 group, respectively (log-rank p < 0.001, Fig. 1). Results were consistent using the approach accounting for the guarantee-time bias, with significantly inferior survival among patients with POD24 compared to the non-POD24 group (Mantel-Byar p < 0.0001, Additional file 1: Figure S1). In the univariate Cox model, factors associated with inferior OS included POD24 status, age, ECOG PS ≥ 2, presence of B symptoms, LDH > ULN, NMZL (compared with EMZL), advanced stage, and R monotherapy rather than immunochemotherapy as first-line treatment (Additional file 1: Table S2). After adjusting for these factors in the multivariable Cox model, POD24 remained associated with significantly inferior OS (HR = 2.50, 95% CI = 1.53–4.09, p = 0.0003, Additional file 1: Table S2). POD24 in the subgroups based on the first-line therapy (R monotherapy or immunochemotherapy, Additional file 1: Figure S2), MZL subtype (Additional file 1: Figure S3), and refractoriness to first-line therapy (Additional file 1: Figure S4) are shown in the Additional file 1.

Twenty-five patients (EMZL = 12, NMZL = 7, and SMZL = 6) experienced histologic transformation (15 in POD24 and 10 in non-POD24 groups), at a median of 3.12 years (range, 0.08–15.58 years) from diagnosis. The cumulative incidence of transformation was significantly higher in the POD24 group compared to the non-POD24 group with the 3- and 5-year rate of transformation being 12% vs 1% and 37% vs 2%, respectively (p < 0.0001, Additional file 1: Figure S5). Most transformation events occurred beyond the initial 24 months of follow-up.

In this multicenter retrospective cohort study, we evaluated the prognostic relevance of POD24 in patients with MZL and made several important observations. First, patients with POD24 had inferior outcomes compared to those without POD24. This was true regardless of the type of systemic therapy received (R monotherapy or immunochemotherapy) at diagnosis. Second, the presence of monoclonal protein at diagnosis and the use of R monotherapy as first-line therapy were associated with POD24. Third, patients with POD24 had a significantly higher risk for histologic transformation compared to those without POD24.

POD24 has emerged as an important prognostic factor in FL treated with first-line chemotherapy. However, in the FL-POD24 study [7], R monotherapy was not included and the relevance of POD24 in FL after R monotherapy is unclear. POD24 was also evaluated as a potential prognostic factor in MZL by Luminari et al. [8]. In that study, 76% of patients received immunochemotherapy and only 9% received R monotherapy. Our study, for the first time to our knowledge, demonstrates the prognostic impact of POD24 for patients treated with R monotherapy in MZL. Many POD24 events led to death also within 24 months suggesting aggressive biology of MZL that relapses within 2 years of starting the initial systemic therapy. Guarantee-time bias is a complex issue that may distort the prognostic assessments when survival between groups is compared with conditioning on events that occur after group assignment (such as POD24) [10]. The initial FL-POD24 study addressed it by counting observation time differently for the POD24 group (from diagnosis) or the non-POD24 group (from the landmark of 2 years after diagnosis) [7]. The prior MZL study, excluded all patients who were censored or died without POD24 before the 24-month landmark [8]. Although we used the same method for compatibility with those prior studies, we note that it is inherently subject to guarantee-time bias because patients in the non-POD24 group “by definition” cannot have an event before 24 months of follow-up (as emphasized in our figures). To overcome this, we reanalyzed the dataset using an approach known to specifically overcome this issue [11], i.e. reassigning the time-at-risk before the POD24 event to the “non-POD24” group regardless of subsequent course (as it is impossible to determine POD24 status before the progression actually occurs). Both approaches showed consistent results, confirming that the prognostic significance of the POD24 event is not an artifact.

The predominant monoclonal protein was IgM followed by IgG in both cohorts (in those with available data). Among the POD24 cohort, 42% (n = 26) of patients with NMZL produced M-protein followed by EMZL (n = 24, 39%) and SMZL (n = 12, 19%). In the non-POD24 cohort, 44% (n = 39) of patients with EMZL produced M-protein followed by SMZL (n = 29, 32%) and NMZL (n = 21, 24%). POD24 is an independent prognostic factor beyond elevated LDH and the presence of B symptoms, which is an indication that it may be capturing a different aspect of biology. The multivariable model suggests that other factors (such as elevated LDH and presence of B symptoms) relate to burden of the lymphoma, while POD24 may be more linked to biology that underpins the resistance to therapy.

Although the patients in the POD24 group had inferior survival compared to the non-POD24 group, the OS in the POD24 group was still good (5-year OS was 75%) in MZL, which is in contrast to FL, where the 5-year OS was only 50% in the POD24 group [7]. However, the rate of transformation in the POD24 group is concerning, and we observed that most histologic transformation events occurred beyond 2 years of follow-up. This important finding underscores the need to report POD24 in future interventional trials in relapsed MZL, especially those evaluating second-line therapies in MZL to help understand if POD24 patients might fare better with non-chemotherapy approaches.

The limitations of the study include the lack of consideration for maintenance therapy, however, in most trials in indolent lymphoma like FL [12] or MZL [2, 13], maintenance rituximab improves PFS without impact on OS. Also, we did not capture the biological correlates such as the presence of complex karyotype, *MALT::BIRC3,* or other common rearrangements specific to MZL, *NOTCH*, *MYD88*, or *TP53* mutation status, etc. which could influence prognosis in MZL or increase the risk of histologic transformation [14–17].

In conclusion, POD24 in MZL might be associated with worse biology and could be used as an additional information point in clinical trials and investigated in translational research as a surrogate of a worse prognosis. Future studies can investigate whether non-chemotherapy approaches could benefit MZL patients with POD24 and whether POD24 after R monotherapy can be salvaged with immunochemotherapy.

## Supplementary Information

**Additional file 1.** Supplementary methods, results, tables and figures.

## Data Availability

Please contact corresponding author for data requests.

## Abbreviations

MZL: Marginal zone lymphoma
POD24: Progression of disease within 24 months
ORR: Overall response rate
CR: Complete remission
PFS: Progression-free survival
OS: Overall survival
