# Supplementary material for: Impact of early relapse within 24 months after first-line systemic therapy (POD24) on outcomes in patients with marginal zone lymphoma: A US multisite study
Source: J Hematol Oncol. 2023 May 8;16:49. doi: 10.1186/s13045-023-01448-y (PMC10165748; doi:10.1186/s13045-023-01448-y)

**SUPPLEMENTAL APPENDIX**

**Table of Contents** 1

**Methods**  2-3

**Results**  3-5

**Factors predictive of POD24:** Table S1 6

**Univariate and multivariable models for OS:** Table S2 7

**Overall Survival – Simon Makuch curves:** Figure S1 8

**OS between POD24 and non-POD24 groups based on first-line therapy:** Figure S2 9

**OS between POD24 and non-POD24 groups based on MZL subtypes:** Figure S3 10

**OS based on the refractoriness to first line therapy in the POD24 group:** Figure S4 11

**Cumulative incidence of transformation between POD24 and non-POD24 groups:** Figure S5 12

**METHODS**

**Patients**

The study was approved by the institutional review boards at all participating sites and was conducted in compliance with the Declaration of Helsinki. Investigators at each site collected data directly from the medical records, according to a standardized protocol.

We collected variables known to be significantly associated with survival outcomes in all subtypes of MZL. Values of laboratory tests (albumin, hemoglobin, serum lactate dehydrogenase [LDH], and beta-2-microglobulin [B2M]) were harmonized according to the upper or lower limit of normal at each institution. All staging procedures (e.g., bone marrow evaluations) and treatment evaluations were conducted according to local practice. The presence of monoclonal paraprotein was ascertained at any time prior to the start of first-line therapy.

**Statistical analysis**

Demographic and disease characteristics were summarized using median and range for continuous variables, and frequency and percentage for categorical variables, compared among study groups using the Wilcoxon signed rank test, and Chi-square test or Fisher’s exact test, respectively. The association between patient characteristics and POD24 was evaluated using the logistic regression model.

Survival analysis according to POD24 was only calculated for patients with at least 24 months of follow-up from the start of initial therapy (unless they experienced progression within those 24 months). Patients who were censored or who died without progression or relapse before 24 months were excluded from the analysis. OS probability was estimated using the Kaplan-Meier method and compared between groups using the log-rank test. Cox proportional hazard regression models were used to estimate the hazard ratios for risk of death. To fully account for guarantee-time bias, we have further compared the OS of patients with/without POD24 event using the split-time approach, without excluding any patients from the dataset regardless of observation time^10^. In this analysis, OS time was measured from the start of first-line therapy for all patients. Observation time for patients with POD24 event was split into pre-POD24 and post-POD24 periods and the time-at-risk prior to progression (for patients who later experienced POD24) was correctly reassigned to the non-POD24 group. This approach produces survival curves (termed Simon-Makuch curves^11^**)** and a log-rank-type test (termed Mantel-Byar test^12^) that are analogous to (left-censored) Kaplan-Meier curves and standard log-rank test yet avoid the guarantee-time bias.

Analyses were performed using SAS, 9.4 (2016 by SAS Institute Inc., Cary, NC, USA), and all statistical tests were two-sided with a type-1 error of <0.05 indicating statistical significance. All estimates were reported with 95% confidence intervals (95%CI).

**RESULTS**

**Patient characteristics**

The median age at the start of systemic therapy was 66 years in the POD24 group compared to 62 years in the non-POD24 group (p=0.003). In this cohort who received first-line systemic therapy, the most common MZL subtype was EMZL (50%), followed by SMZL (26%) and NMZL (24%). The proportion of NMZL was higher (32%) in the POD24 group compared to 21% in the non-POD24 group (p=0.04). Compared to the non-POD24 group, patients in the POD24 group had significantly more often ECOG PS of ≥2 (7% vs 15%, p=0.03), serum LDH higher than the institutional baseline (24% vs 36%, p=0.01), low albumin (14% vs 22%, p=0.03), presence of monoclonal protein (27% vs 47%, p<0.0001), and they more often received R monotherapy as first-line therapy (66% vs 53%, p=0.02). The median DTI was 1.8 months (1.6 months in the POD24 and 1.9 months in the non-POD24 group). The most common immunochemotherapy regimen was R-bendamustine (n=136) followed by R-CHOP (n=35) and R-CVP (n=23).

**Subgroup analysis**
Considering disparate survival related to the type of first-line therapy, we further investigated the prognostic value of POD24 in groups treated with R monotherapy or immunochemotherapy separately. In both subgroups, the POD24 group had significantly inferior OS compared to the non-POD24 group. The 3- and 5-year OS estimates for those receiving R were 82% (95%CI, 73-89%) and 74% (95%CI, 62-83%) in POD24 compared to 96% (95%CI, 92-98%) and 90% (95%CI, 84-94%) in non-POD24 (p=0.003, **Figure 2A**), while the 3- and 5-year OS estimates for those receiving immunochemotherapy were 83% (95%CI, 68-92%) and 75% (95%CI, 58-86%) in the POD24 compared to 97% (95%CI, 93-99%) and 94% (95%CI, 87-97%) in the non-POD24 groups, respectively (p=0.001, **Figure 2B**).

Patients with EMZL had significantly inferior OS when they experienced POD24 compared to the non-POD24 group, which is in line with the main analysis (p<0.0001, **Figure S3A**). The direction of association was consistent in the NMZL (**Figure S3B**) and SMZL (**Figure S3C**) subgroups but did not reach statistical significance.

We also evaluated the outcomes of patients based on the refractoriness to first line therapy in the POD24 group. Primary refractory disease was defined as patients who had no response or progression of disease at the end of first-line therapy or within 6 months of treatment completion. Not surprisingly, those with primary refractory disease had worse outcomes compared to those without primary refractory disease (p=0.01) among patients with available information (n=115, **Figure S4**).

**Causes of Death**
There were 36 deaths related to lymphoma (36/78, 46%). Among these 18 were in the POD24 (18/34, 53%) and 18 in the non-POD24 group (18/44, 41%), that was not significantly different among the two groups.

**Table S1. Multivariable logistic regression to identify factors predictive of POD24**

| **Variables** | **OR** | **95%CI** | **p-value** |
| --- | --- | --- | --- |
| Age | 1.01 | 0.99 - 1.03 | 0.28 |
| Sex |  |  |  |
| Female | Referent |  |  |
| Male | 0.90 | 0.57 - 1.43 | 0.67 |
| BMI |  |  |  |
| <30 | Referent |  |  |
| ≥30 | 0.70 | 0.42 - 1.17 | 0.17 |
| MZL subtype |  |  |  |
| EMZL | Referent |  |  |
| NMZL | 1.80 | 1.00 - 3.25 | 0.05 |
| SMZL | 0.86 | 0.46 - 1.58 | 0.62 |
| ECOG PS |  |  |  |
| 0-1 | Referent |  |  |
| ≥2 | 1.89 | 0.92 - 3.87 | 0.08 |
| Stage |  |  |  |
| 1-2 | Referent |  |  |
| 3-4 | 1.39 | 0.78 - 2.50 | 0.27 |
| B Symptoms |  |  |  |
| No | Referent |  |  |
| Yes | 1.07 | 0.60 - 1.91 | 0.82 |
| First-line Treatment |  |  |  |
| Rituximab alone | Referent |  |  |
| R-chemotherapy | 0.48 | 0.29 - 0.80 | **0.005** |
| Monoclonal Protein at dx |  |  |  |
| No | Referent |  |  |
| Yes | 2.87 | 1.70 - 4.85 | **<.0001** |
| LDH >ULN |  |  |  |
| No | Referent |  |  |
| Yes | 1.51 | 0.91 - 2.50 | 0.11 |

Abbreviations: BMI- body mass index, ECOG PS- Eastern Cooperative Oncology Group performance status, LDH- lactate dehydrogenase, ULN- upper limit of normal, dx-diagnosis, OR-odds ratio

**Table S2. Univariate and multivariable models for Overall Survival**

| **Variable** | **Univariate** | | **Multivariable** | |
| --- | --- | --- | --- | --- |
|  | **HR (95% CI)** | **p-value** | **HR (95% CI)** | **p-value** |
| POD24 status |  |  |  |  |
| Non-POD24 | 1.00 |  | 1.00 |  |
| POD24 | 2.66 (1.69, 4.17) | **<.0001** | 2.50 (1.53, 4.09) | **0.0003** |
| Age | 1.06 (1.04, 1.09) | **<.0001** | 1.07 (1.04, 1.10) | **<.0001** |
| ECOG PS |  |  |  |  |
| 0-1 | 1.00 |  |  |  |
| ≥2 | 2.78 (1.57, 4.92) | **0.0004** |  |  |
| B Symptoms |  |  |  |  |
| No | 1.00 |  |  |  |
| Yes | 2.26 (1.39, 3.66) | **0.0009** | 2.12 (1.25, 3.61) | **0.006** |
| LDH >ULN |  |  |  |  |
| No |  |  |  |  |
| Yes | 2.19 (1.35, 3.56) | **0.002** | 2.09 (1.27, 3.44) | **0.004** |
| Monoclonal protein |  |  |  |  |
| No |  |  |  |  |
| Yes | 1.23 (0.74, 2.07) | 0.41 |  |  |
| MZL subtype |  |  |  |  |
| EMZL | 1.00 |  |  |  |
| NMZL | 1.87 (1.08, 3.23) | **0.02** |  |  |
| SMZL | 1.67 (0.97, 2.88) | 0.06 |  |  |
| Stage |  |  |  |  |
| 1-2 | 1.00 |  |  |  |
| 3-4 | 2.64 (1.36, 5.15) | **0.004** |  |  |
| First-line Treatment |  |  |  |  |
| Rituximab alone | 1.00 |  |  |  |
| R-chemotherapy | 0.58 (0.35, 0.95) | **0.03** |  |  |

Abbreviations: LDH- lactate dehydrogenase, ULN- upper limit of normal, UVA- univariate analysis, MVA- multivariable analysis

**Figure S1. Overall Survival – Simon-Makuch curves**


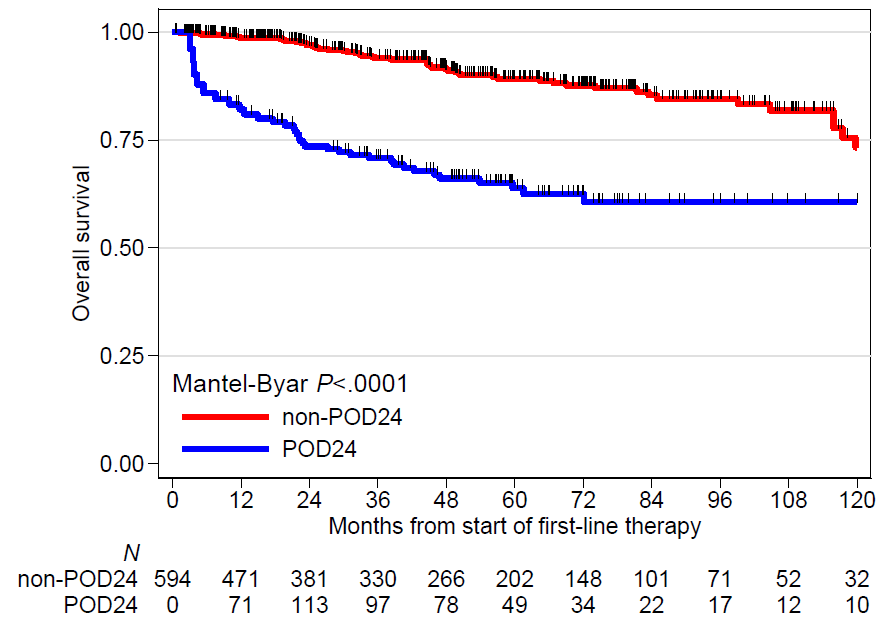


**Figure S2. Overall Survival between POD24 and non-POD24 groups based on first-line therapy A) Rituximab monotherapy B) R-chemotherapy**


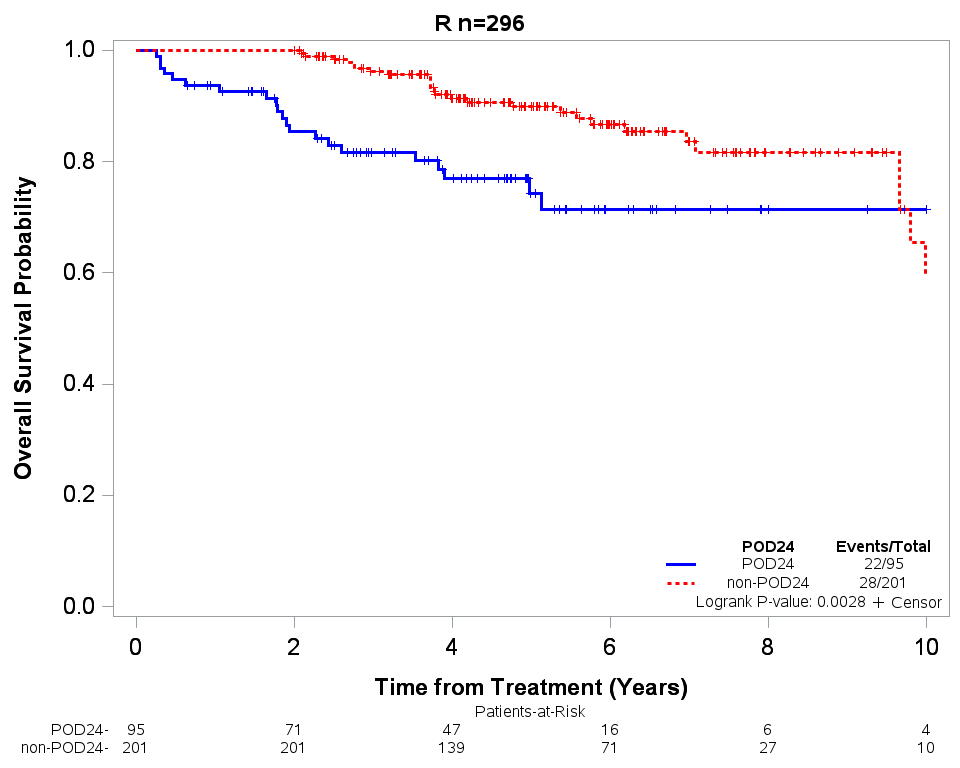


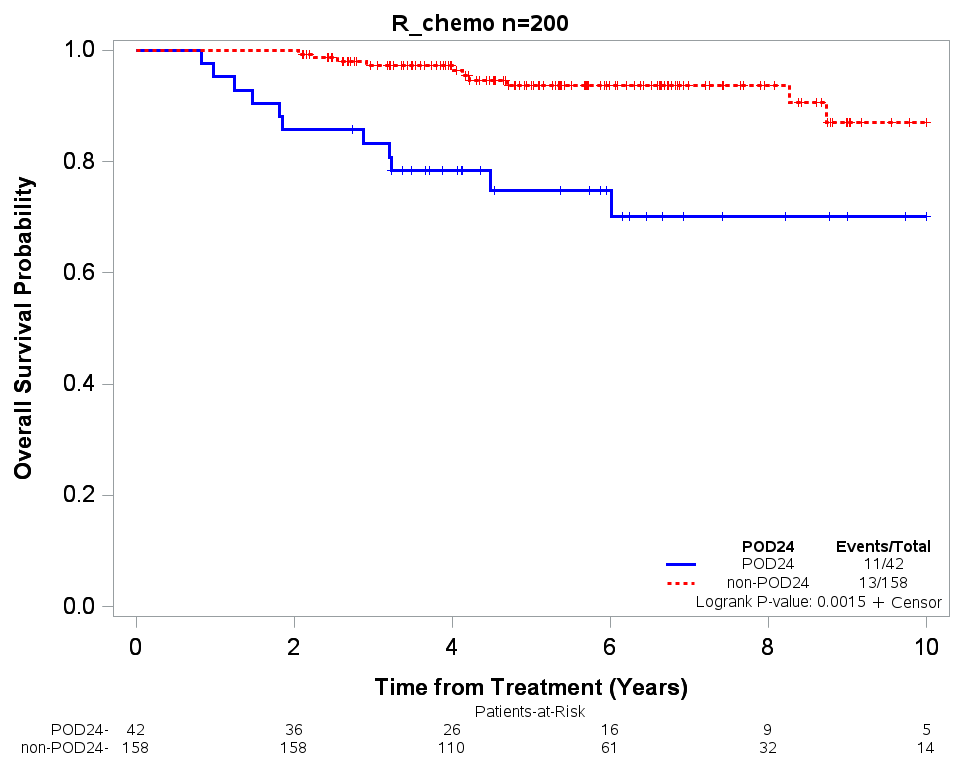


**Figure S3. Overall Survival between POD24 and non-POD24 groups based on MZL subtypes**


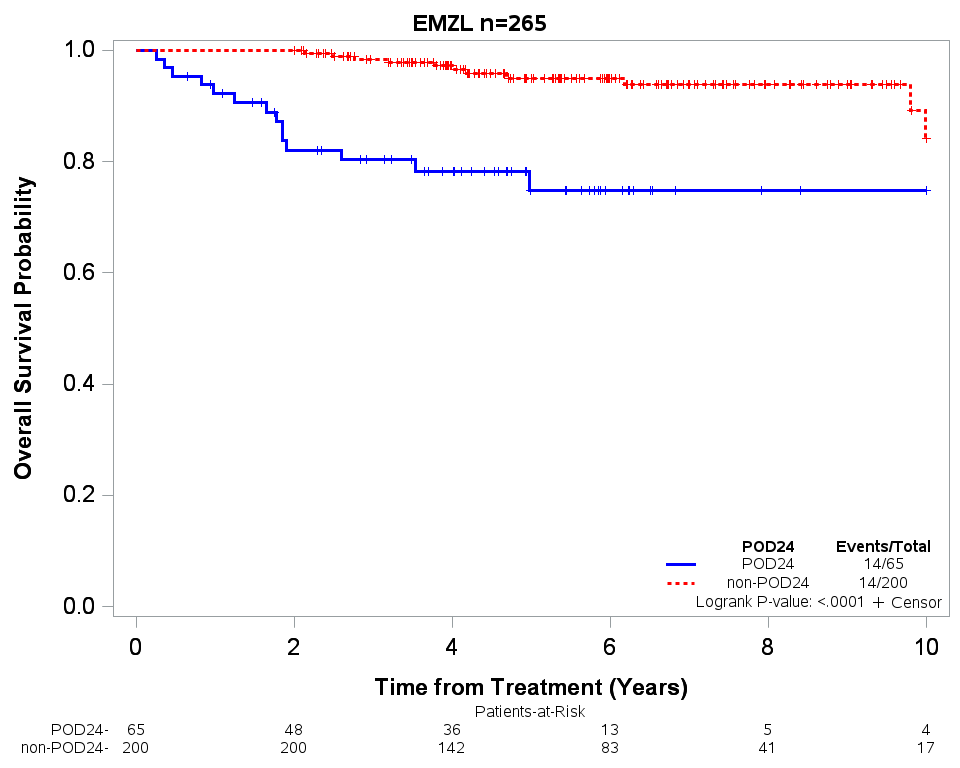

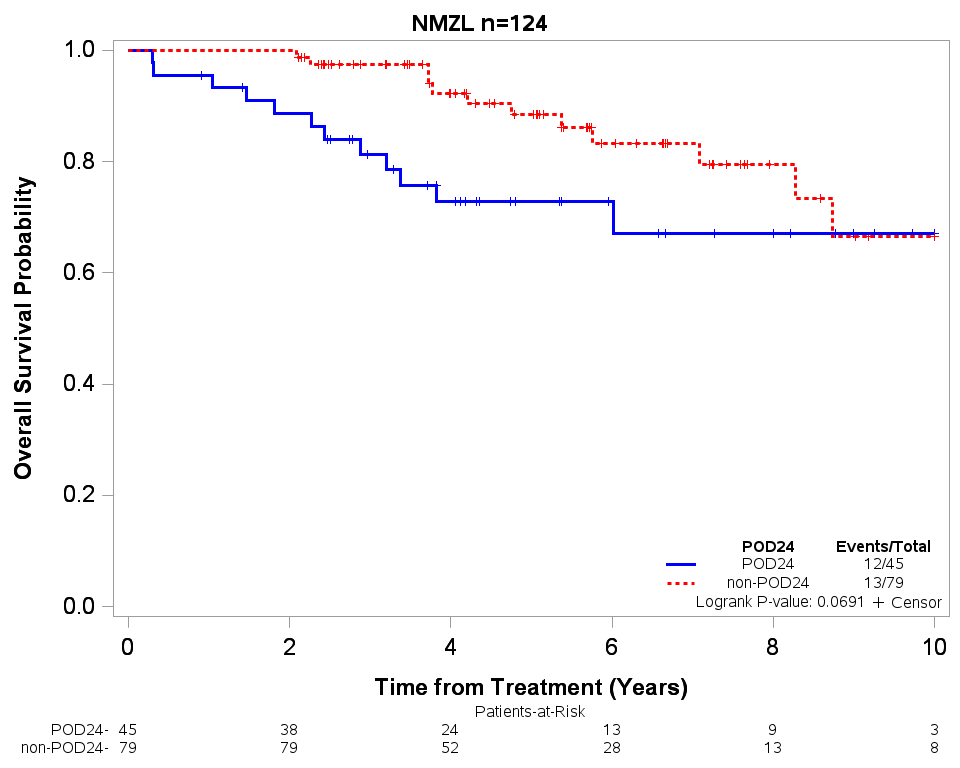


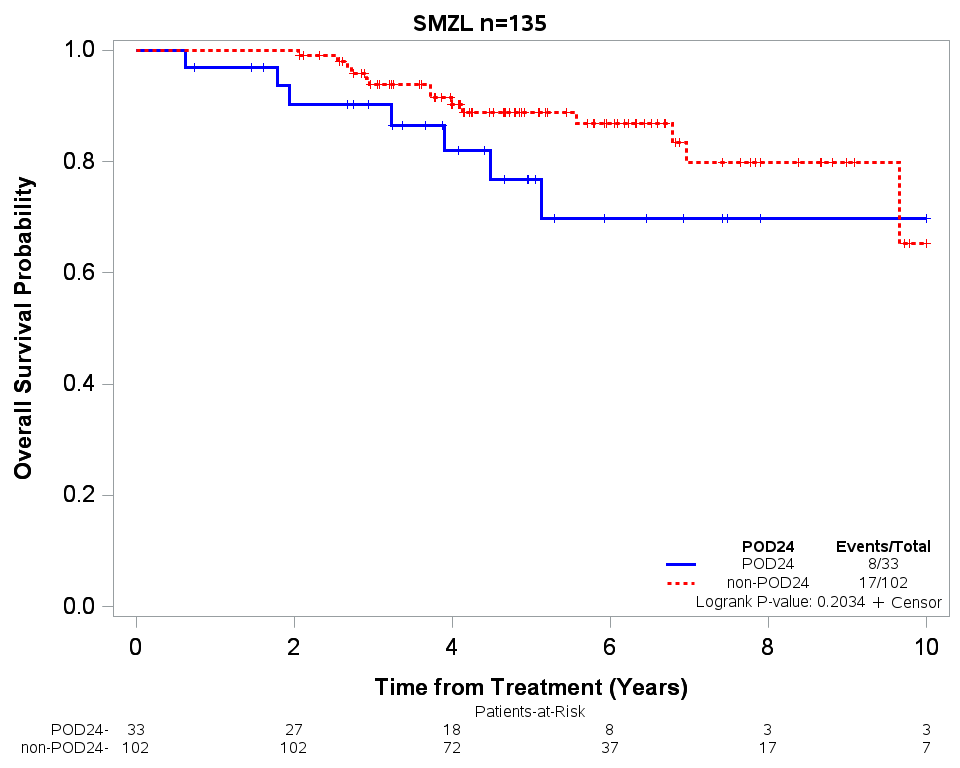


**Figure S4. Overall Survival based on the primary refractory status among the patients in the POD24 cohort**


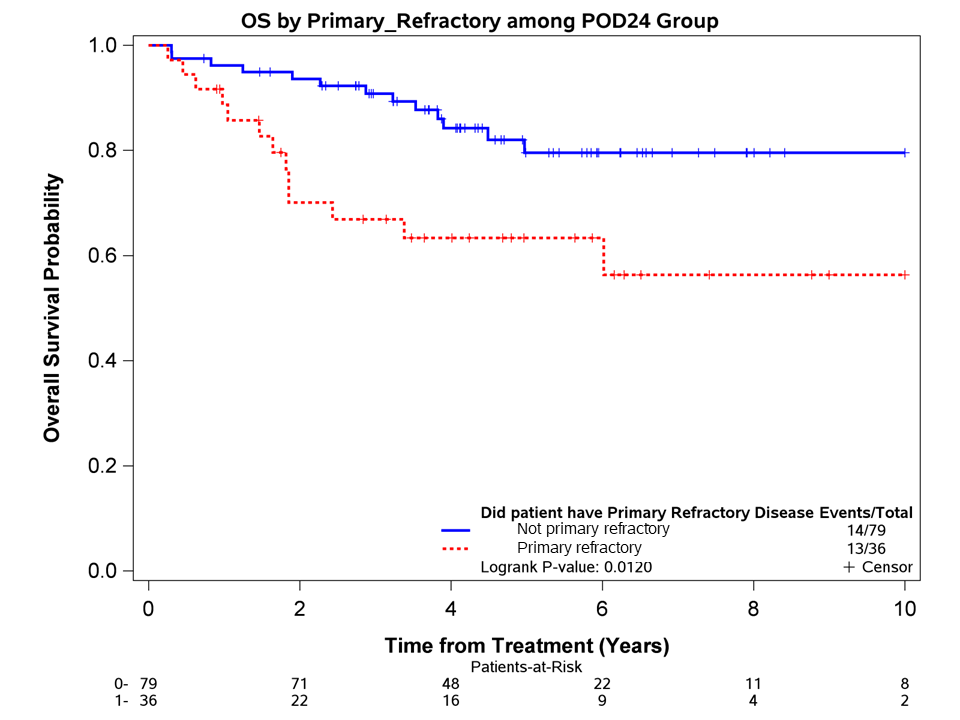


**Figure S5. Cumulative incidence of histological transformation between POD24 and non-POD24 groups**


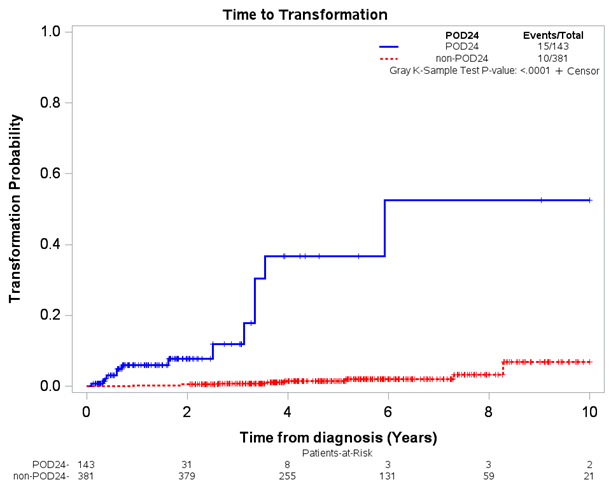

Supplement: Supplementary file 1 — Additional file 1. Supplementary methods, results, tables and figures. [file 13045_2023_1448_MOESM1_ESM.docx]
